# Healthier Oils: A New Scope in the Development of Functional Meat and Dairy Products: A Review

**DOI:** 10.3390/biom13050778

**Published:** 2023-04-30

**Authors:** Carmen Botella-Martínez, José Ángel Pérez-Álvarez, Estrella Sayas-Barberá, Casilda Navarro Rodríguez de Vera, Juana Fernández-López, Manuel Viuda-Martos

**Affiliations:** IPOA Research Group, Agro-Food Technology Department, Centro de Investigación e Innovación Agroalimentaria y Agroambiental (CIAGRO-UMH), Miguel Hernández University, 03312 Orihuela, Spain

**Keywords:** reformulation, vegetable oils, marine oils, pre-emulsification, microencapsulation, gelled emulsion, oleogels, meat products, dairy products

## Abstract

In the present day, it has been widely established that a high intake of animal fat that contains a high content of saturated fatty acids may cause several life-threatening diseases, including obesity, diabetes-type 2, cardiovascular diseases, as well as several types of cancer. In this context, a great number of health organizations and government agencies have launched campaigns to reduce the saturated fat content in foods, which has prompted the food industry, which is no stranger to this problem, to start working to develop foods with a lower fat content or with a different fatty acid profile. Nevertheless, this is not an easy task due to the fact that saturated fat plays a very important role in food processing and in the sensorial perception of foods. Actually, the best way to replace saturated fat is with the use of structured vegetable or marine oils. The main strategies for structuring oils include pre-emulsification, microencapsulation, the development of gelled emulsions, and the development of oleogels. This review will examine the current literature on the different (i) healthier oils and (ii) strategies that will be potentially used by the food industry to reduce or replace the fat content in several food products.

## 1. Introduction

Non-communicable diseases are the principal cause of death around the world. Globally in 2019, non-communicable diseases were responsible for 73.6% of deaths, which is higher than all other causes combined [1]. Among non-communicable diseases, cardiovascular diseases account for the highest number of deaths [2]. In this sense, out of the 17 million premature deaths (under the age of 70) due to non-communicable diseases in 2019, 38% were caused by cardiovascular diseases. Cardiovascular disease comprises coronary heart disease (heart attacks), cerebrovascular disease (strokes), heart rhythm problems (arrhythmias), and pulmonary embolism, among others [3].

Atherosclerosis, which is linked to dyslipidaemias and obesity, is the principal risk factor in the development of heart attacks and ischemic strokes [4]. Atherosclerosis can be considered a multifactorial disease in which modifiable factors, including tobacco smoking, alcohol consumption, no physical activity, bad dietary habits including high intake of salt and saturated fats, and a deficiency in the consumption of vegetables and fruit [5,6], and non-modifiable factors, such as age, gender, ethnicity, and family history of atherosclerosis, interfere in its development [7].

Among the modifiable factors, saturated fat intake reduction is the principal issue that can be addressed. Meat, meat products, and dairy products are the main sources of saturated fat. In this sense, in the last decade a huge number of countries have applied diverse initiatives, including food labelling schemes, healthy eating promotion campaigns, risk assessment measures, and trade consultations, to reduce the intake of foods with a high saturated fat content [8]. Thus, the World Health Organization recommends that the total fat consumption must be lower than 30% of total energy intake (TEI), the saturated fat consumption must be lower than 10% of TEI, and the total *trans*-fats consumption must be lower than 1% of TEI, with the ultimate objective of replacing saturated fats and *trans*-fats with mono- and polyunsaturated fats [9].

However, it is important to highlight that in the organism, fat not only plays a role as an energy source but also plays a very important role as (*i*) a structural component of the body (*ii*) in the transport of fat-soluble vitamins as well as (*iii*) through intervening in physiological processes of the organism. Additionally, it is essential for the correct functioning of a series of biological functions during growth and development [10,11]. In food products, fat has both technological and sensory functions. Regarding the technological functions, fat content is related to an increase in emulsion stability, a reduction of cooking loss, and the regulation of the drying process in dry-cured products, besides exerting a great influence on the rheological and structural properties [12,13]. In reference to sensorial properties, fat improves the palatability of products, improves the overall texture of products, increases juiciness, decreases hardness, enhances the color properties of products, and increases the flavor of products [13,14,15]. For these reasons, reducing or eliminating fat from food, especially meat and dairy products, is time-consuming and labor-intensive. The agri-food industry, together with the scientific community, has already started to look for alternatives to replace saturated fat with other types of fat that have better fatty acid profiles that are rich in monounsaturated and polyunsaturated fatty acids. Thus, low-fat reformulated products with better fatty acid profiles are increasingly available on the market.

The principal sources of fat rich in monounsaturated and polyunsaturated fatty acids are vegetable (seeds and fruits) and marine (fish and seaweed) oils [16]. Due to their composition, these fats are more susceptible to rancidity, which leads to a loss of nutritional and sensory value of the product, as well as a reduction in shelf life, making their use in the preparation of meat or dairy foods more difficult [17]. These side effects may be reduced using diverse strategies that could stabilize the system to avoid oil separation in the food matrix [18]. These strategies include the transformation of liquid oils obtained from vegetables, seaweeds, or fishes into solid-like fats without the formation of artificial *trans*-fat [15]. The main techniques for structuring oils and improving their technological and functional properties include pre-emulsification, microencapsulation, the development of gelled emulsions, and the development of oleogels [13,19,20,21].

**Figure 1:**
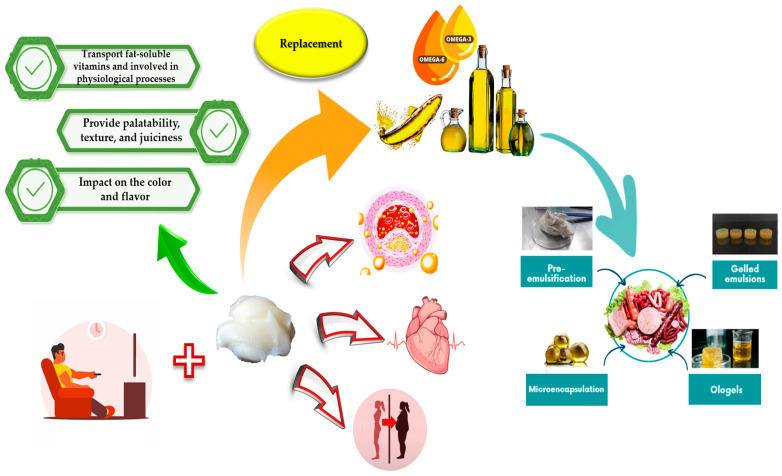
Effects of animal fat in the body and the main strategies to replace it in food products.

In the scientific literature, it is possible to find a high number of works that have informed of the use of pre-emulsification, microencapsulation, oleogels, and gelled emulsions prepared with vegetable or marine oils (with a high content of polyunsaturated fatty acids) as saturated fat replacers in several foods, including meat and dairy products [22,23,24,25,26] (Figure 1).

The aim of this work is to examine the current literature on different (*i*) healthier oils and (*ii*) strategies that will be potentially used by the food industry to reduce or replace the fat content in several types of food products.

## 2. Healthier Oils

### 2.1. Vegetable Oils

Global vegetable oil production has increased in the last few years to reach 217.7 million metric tons in 2022 [27]. An important feature common to most vegetable oils is the high percentage of unsaturated fatty acids in triacylglycerols. Although this fact is beneficial for health, deep frying vegetable oils for a longer period, as occurs with saturated fats from animal origin, converts them into compounds responsible for off-type flavors such as short-chain hydroperoxides, aldehydes, and keto derivatives [28]. Table 1 shows the lipid profile of some edible vegetable oils.

#### 2.1.1. Oils from Oilseeds

Oilseed plants are one of the largest crop groups in world production because their seeds have a high percentage of high-quality fatty acids and proteins. Among the most commonly used seeds for the extraction of edible oils are soybeans, rapeseed (canola), sunflower, cottonseed, corn, and peanut, among others. Oils from rapeseed, soybeans, and sunflower seeds account for 87% of global vegetable oil production [27]. They are rich in ω-3 and ω-6 fatty acids, vitamins A, D, E, and K, and minerals such as zinc, calcium, magnesium, potassium, copper, and iron. Most of them are principally obtained by solvent (particularly *n*-hexane) extraction or mechanical expellers. Once the oils are extracted from the seeds, they need a refining process (compulsory if they are chemically extracted) prior to consumption to improve the conservation and nutritional conditions, since some seeds contain a series of substances called antinutrients that can be toxic.

##### Soybean Oil

Soybean oil is extracted from the seeds of the soybean (*Glycine max*). The composition of soybean oil is 14–16% saturated fatty acids (10% of palmitic acid (16:0) and 4% of stearic acid (18:0)), 20–24% monounsaturated fatty acids (mainly oleic acid (18:1) approx. 18–20%), and 62–66% polyunsaturated fatty acids (54% of linoleic acid (18:2) and 12% linolenic acid (18:3)) [29,30]. Like other vegetable oils, soybean oil also contains phytosterols (β-sitosterol, campesterol, stigmasterols, etc.), tocopherols (α-tocopherol and β-tocopherol), β-carotene, lutein, and chlorophylls [30].

##### Canola Oil

Canola oil is produced from rapeseed (mainly *Brassica napus* L). This oil exhibits the best fatty acid composition among all common oils. It has the ideal combination of the lowest level of saturated fatty acids (7–8%), a high content of monounsaturated fatty acids (63–66%), and an excellent ratio of ω-6 (linoleic acid) to ω-3 (α-linolenic acid) polyunsaturated fatty acids (19.0/9.1 g/100 g) [31]. This composition is responsible for the health properties associated with canola oil consumption. Among the non-glyceridic content, canola oil also contains phytosterols (mainly β-sitosterol) and vitamins E (as α and β-tocopherol) and K [32].

##### Sunflower Seed Oil

Sunflower oil is extracted from the seeds of sunflowers *(Helianthus annuus)*. The main fatty acid fraction is polyunsaturated fatty acids (44–75% linoleic acid), followed by monounsaturated fatty acids (22–24% oleic acid) and saturated fatty acids (15%: 7% palmitic acid and 8% stearic acid). It is also a rich source of vitamin E [33]. Depending on plant breeding and industrial processing, four types of sunflower oil have been obtained: high-linoleic (69% linoleic acid), high-oleic (82% oleic acid), mid-oleic (65% oleic acid), and high-stearic combined with high-oleic (18% stearic acid and 72% oleic acid) [34]. However, from these four types of sunflower oil, the most used are the high-oleic and the mid-oleic sunflower oils.

##### Cottonseed Oil

Cottonseed oil is extracted from the seeds of cotton plants from various species, mainly *Gossypium hirsutum* and *Gossypium herbaceum*. It has been reported that yield, nutritional value, and chemical composition seed oils are affected by several factors, mainly including genetic constitution [59]. The main fatty acid fraction in cottonseed oil is polyunsaturated fatty acids (52–55%; mainly linoleic acid 52% and a small amount of (<1%) linolenic acid), followed by saturated fatty acids (25–30%; palmitic acid 26% and stearic acid 2%) and monounsaturated fatty acids (18%, oleic acid) [35].

##### Corn Oil

Corn oil is extracted from the germ of the seeds of the corn plant (*Zea mays* L.), and for this reason it is commonly known as “corn germ oil.” The germ represents between 9–11% of the seed weight and contains about 80% of the lipids found in the whole seed [60].

Corn oil belongs to the group of vegetable oils with high levels of linoleic and oleic acids (similar to sunflower oil). Specifically, it has been reported that about 60% of its fatty acids are polyunsaturated fatty acids (52% linoleic acid and only 1% of linolenic acid), 25% are monounsaturated fatty acids (oleic acid), and 15–17% are saturated fatty acids (palmitic acid as the predominant one) [37,38]. Corn oil also represents an important source of minor bioactive lipids, such as phytosterols (β-sitosterol 55–67%, campesterol 19–24%, stigmasterol 4–8%, and Δ-5-avenasterol 4–8%), tocopherols, tocotrienols, and carotenoids (especially xantophylls, lutein, and zeaxanthin) [61].

##### Peanut Oil

Peanut oil is the oil from the seed (peanut) of the peanut plant (*Arachis hypogaea*). It is predominantly perceived as a valuable source in relation to edible-oil along with the protein source that mainly remains in the peanut cake after oil extraction [62]. Peanut oil has oleic acid as the predominant fatty acid, accounting for 48 to 57%, with a mean value of 53%. In addition, it contains linoleic acid, accounting for 27 to 38%, with a mean value of 32%. Peanut oil also contains appreciable amounts of saturated fatty acids (10–15%), especially palmitic (8–11%) and stearic (2–4%) acids [39]. Like other oilseeds, medium and high oleic peanut varieties comprising 66–69% and 78–80% of monounsaturated fatty acids, respectively, have been developed [40,41]. Peanut oil contains a considerable amount of phytosterols (207 mg/100 g), which is even greater than olive oil’s phytosterol level [63]. In addition, peanut oil provides a valuable source of lipid-soluble vitamins, such as tocopherols (Vitamin E) and pantothenate [64].

##### Walnut Oil

Walnut oil is generally extracted by cold pressing from the fruit of the walnut tree (*Juglans regia*). Walnut seeds generally contain between 52% and 70% of oil, which depends on the cultivar [65]. In particular, walnut oil contains between 2.4–5.3% palmitic acid, between 1.4–4.1% stearic, between 17.66–20.7% oleic acid, between 48.50–53.204% linoleic acid, and between 13.7–15.90% linolenic acid [66,67]. Walnut oil also contains a great number of phytosterols (106.5 mg/100 g) than others seed oils [68]. Additionally, walnut oil has a high content of α-, β-, γ-, and δ-tocopherols (Vitamin E) and pantothenate [69].

##### Chia Oil

Chia (*Salvia hispanica* L.) is native to Central America. The oil content in chia seeds is about 34%, which presents the highest percentage of α-linolenic acid known so far (62–64%) as well as the highest content (82.3%) of essential fatty acids (α-linolenic acid and linoleic acid) [70]. The α-linolenic acid constitutes more than 60% of all total fatty acids in chia seeds, making this product one of the most important sources of α-linolenic acid in our diet [43]. Moreover, chia oil has the additional advantage of having a low content of saturated fatty acids; in particular, the oil contains 6.9% palmitic acid and 2.8% stearic acid [70].

##### Oil from Non-Traditional Oilseeds

Recently, it is possible to find oils obtained from seeds that are not usually cultivated for this purpose, but which are attracting increasing interest due to the special fatty acid compositions of the oils obtained. Regarding this, hemp oil (extracted from the seeds of *Cannabis sativa* L.) contains more than 80% of polyunsaturated fatty acids, including essential fatty acids usually not contained in traditional oils used for the human diet, consisting mainly of *w*-6 linoleic acid (50–55%) and *w*-3 α-linolenic acid (13–15%) [44]. In addition, the *w*-3/*w*-6 ratio is 3:1, which agrees with European Food Safety Agency recommendations [71]. Its unsaponifiable fraction is also a source of interesting minor bioactive compounds such as tocopherols, vitamins D and E, and phytosterols [45]. Linseed oil (*Linum usitatissimum* L.) is an important component in the development of functional foods, as it is rich in polyunsaturated fatty acids and phenolic compounds [72]. Linseed oil contains a low amount of saturated fatty acids (around 9% of total fatty acids), a moderate content of monounsaturated fatty acids (around 18%), and a high amount of polyunsaturated fatty acids (73%) [73]. Date seed oil, extracted from the seeds of the fruit of date palm, is liquid at room temperature, yellowish in color, and has a pleasant odor. This oil is considered a source of oleic acid (41–48%), with an important amount of saturated fatty acids (45–50%, mainly lauric acid 19%) and small amounts of polyunsaturated fatty acids (8%, mainly linoleic acid) [46]. It is also a good source of tocopherols and tocotrienols (74 mg/100 g), phytosterols (mainly β-sitosterol, campesterol, and Δ5-avenasterol), and polyphenols, containing an even higher amount of polyphenols than olive oil [74].

#### 2.1.2. Oils from Fruits

##### Olive Oil

Olive oil is extracted from the fruit of the olive tree (*Olea europaea* L.), which was one of the first plants to be cultivated for oil production. The main fatty acid in olive oil is oleic acid (65–85%), accounting for 55–83% of total fatty acids. It also contains variable amounts of linoleic acid (3–21%) and linolenic acid (<1%), with small saturated fatty acids contents (8–13%) [31,47]. Concerning bioactive compounds, their main representatives are the same of oil in general, namely tocopherols and phenolic compounds such as hydroxytyrosol and oleuropein, but also other compounds as pigments, such as provitamin A compounds and chlorophylls [48].

##### Coconut Oil

Coconut oil is an edible oil derived from the wick, meat, and milk of the fruit of the coconut palm (*Cocos nucifera*). The oil is a rich source of saturated fatty acids (85–90%), with short- and medium-chain fatty acids accounting for 70% of these fatty acids. The predominant fatty acid is lauric acid, representing 44–50% of the total fatty acids. It has a low content of unsaturated fatty acids (6–11%), oleic acid being the majority (5–8%), with a negligible content of both *w*-6 and *w*-3 polyunsaturated fatty acids and a low *w*-6/*w*-3 ratio (<4) [49].

##### Avocado Oil

Avocado oil is one of few edible oils not derived from seeds; it is extracted from the pulp of avocados, the fruit of *Persea Americana*, with an oil content of about 60%. Avocado oil has a similar monounsaturated fat profile (>60%) to olive oil. It can contain up to 71% of monounsaturated fatty acids (61% oleic acid and 10% palmitoleic acid), 13% of polyunsaturated fatty acids (12% linoleic acid and 1% linolenic acid), and 16% of saturated fatty acids (15% palmitic acid and less than 1% of stearic acid) [31,51]. Avocado oil has a high concentration of phytosterols (3.3 g to 4.5 mg/g of oil, higher than in olive oil), of which the most abundant is β-sitosterol, followed by sitostanol, cycloartenol, cycloeucalenol, and D7-avenasterol [75].

### 2.2. Oils from Marine Origin

#### 2.2.1. Seaweed Oils

Seaweed oil is extracted from seaweed (also known as macroalgae or marine algae) and can be considered an alternative source of edible oils. Although its lipid content is low (0.1–10%), its use has raised considerable interest in recent years due to its high polyunsaturated fatty acids content (15–30%), specifically α-linolenic (*w*-3), octadecatetraenoic (*w*-3), arachidonic (*w*-6), and eicosapentaenoic acids (*w*-3) [76]. However, the saturated fatty acid fraction is the main fraction (45–55%), of which more than half consists of palmitic acid (C16). The monounsaturated fatty acids content (19–25%) is a little less than that of the polyunsaturated fatty acids (25–40%) [52]. It must be noted that the lipid fatty acid composition depends on the type of algae [53]. Red seaweed species (*Rhodophyta*) contain significant amounts of polyunsaturated fatty acids. Their two main polyunsaturated fatty acids are eicosapentaenoic (C20:5 *w*-3) and arachidonic acids (C20:4, *w*-6), and they also have a high oleic acid content. Brown seaweeds (*Phaeophyta*) show the highest relative concentration of monounsaturated fatty acids. Green seaweeds (*Chorophyta*) are characterized by C16 and C18 polyunsaturated fatty acids, with a high C18/C20 polyunsaturated fatty acids ratio and a high degree of unsaturation. Unlike red and brown algae, green algae contain large amounts of hexadecatrienoic (16:3) and hexadecatetraenoic (16:4) polyunsaturated fatty acids and high contents of C18 polyunsaturated fatty acids (C18:2 and C18:3). Linoleic acid is the main polyunsaturated fatty acid found in most chlorophytes and α-linolenic acid is the main polyunsaturated fatty acid found in Ulvales algaes [54]. Algae oil is also rich in some bioactive compounds found in its unsaponifiable fraction, mainly phytosterols, tocopherols, and carotenoids [52].

#### 2.2.2. Fish Oils

Fish oil is derived from the tissues of fish (liver in lean fish and flesh in fatty fish). The main sources of fish oil are pelagic species caught in large quantities, particularly those with oily flesh (salmon, tuna, mackerel, and herring) or small fish (anchovies and capelin). Like seaweed oil, fish oil is characterized by a high level of the very long-chain, highly unsaturated ω-3 fatty acids eicosapentaenoic acid (C20:5, ω-3), docosapentaenoic acid (C22:5, ω-3), and docosahexaenoic acid (C22:6, ω-3). The growing awareness about the importance of ω-3 fatty acids in nutrition and health has led to a significant increase in fish oil consumption. The global fish oil market size is estimated to reach 284,412 million dollars by 2027, with a compound annual growth rate of 5.79% from 2021 to 2027 [55].

The lipid content in seafood species varies between 0.3–20%, depending on factors such as species, nutrition, geographical region, season, biological condition, age, gender maturity, reproduction, and temperature [56]. Total saturated fatty acids, monounsaturated fatty acids, and polyunsaturated fatty acids percentages of the total lipids range from 28% to 37%, from 18% to 38%, and from 11% to 35%, respectively. Palmitic acid is a dominant saturated fatty acid, followed by stearic acid. Palmitoleic and oleic acids are the main monounsaturated fatty acids (Table 1). The ω-3 polyunsaturated fatty acids eicosapentanoic and docosahexaenoic fatty acids are predominant [77], and therefore fish oil is an important dietary source of these essential fatty acids.

### 2.3. Insect Oils

Edible insects have been revealed as a sustainable and alternative source of nutrients, mainly proteins (trying to address human food demand), but also lipids, which are the second largest fraction and are sometimes regarded as a co-product. Lipid content in insects ranges between 10–35% in dry matter. Both lipid content and fatty acid composition can vary depending on species, state of growth, and extraction technologies, among other factors. The lipid content of *T. molitor* in the larvae state is 33%, clearly much higher than the content reported for the adult states of other insects (15% for *A. domesticus*) [57]. Most insect lipids are liquid at room temperature, which indicates that they are rich in UFA. The oil from *T. molitor* has a light-yellow color; oleic acid is the predominant fatty acid (32–38%), followed by linoleic acid (*w*-6, 20–25%) and palmitic acid (18–23%). The fatty acid profile of *A. domesticus* lipids is similar to that of *T. molitor* [58], but in this case the proportion of linoleic acid is higher than that of oleic acid [57] (Table 1).

## 3. Strategies for Structuring Oils

**Figure 2:**
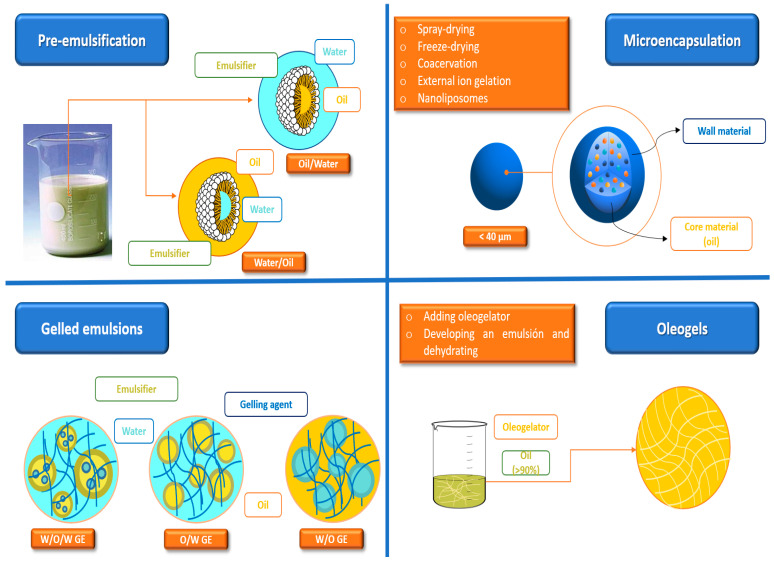
The main strategies to structure vegetable or marine oils.

As mentioned above, beyond esterification or hydrogenation, there are many ways of structuring oils for their subsequent incorporation into food matrices. Additionally, the strategies attempt to mimic the appearance, plasticity, and rheological properties of animal fat in addition to improving nutritional quality and the lipid profile [13,78]. In this regard, as shown in Figure 2, pre-emulsification, oil encapsulation, oleogels, and gelled emulsions are several varieties of solid oil structured systems that offer promising results as fat substitutes in the development of healthier foods [79,80,81,82,83,84].

Table 2 presents several studies where vegetable and marine oils were structured using different strategies to be applied in foods.

### 3.1. Pre-Emulsification

Pre-emulsification of oils with a high content of monounsaturated and polyunsaturated fatty acids can be considered a good method to carry them into several food matrices such as meat, bakery, and dairy products without reducing their technological and physicochemical properties [79,80]. To obtain this structure, the oils must be blended with different emulsifiers, mainly proteins, including whey protein, lecithin, soy proteins, and caseinates, among others [81], or with carbohydrates such as cellulose ethers [85]. The methodologies utilized to elaborate the pre-emulsified oils, the emulsifiers selected, as well as the concentrations of oil, water, and protein used are very different among the studies present in the scientific literature, making it very difficult to standardize a process. Thus, Bolger et al. [79] carried out a study on pre-emulsified flaxseed oil and vitamin E to be used as a fat replacer in low-fat chicken sausages. For the study, one part of soy protein concentrate was mixed with four parts of ice water with a hand-blender for 1 min. Then four parts of flaxseed oil were added and mixed for 5 min. To elaborate a low-fat pork patty, Lee et al. [12] prepared an oil-in-water emulsion by mixing water (70%), tween 80 (3.5%), canola oil (30%), and lecithin (1%) in a homogenizer at 4000 rpm for 3 min. In a similar study, Urgu-Öztürk et al. [81] designed a healthier beef sausage elaborated with a pre-emulsion made by emulsifying hazelnut oil (51.6%) with water (42.1%), using sodium caseinate (5.3%) and sodium chloride (1%) as emulsifier agents, and using a food processor at 5800 rpm for 2 min. In the same sense, to elaborate low-fat cooked lamb sausages de Carvalho et al. [101] made three pre-emulsions with chia, olive, and linseed oil, water, and sodium caseinate as an emulsifying agent in a proportion of 5:5:1 in a homogenizer at 5000 rpm for 3 min. Li et al. [102] made a pre-emulsion for reduced-fat filling in steamed buns. For that, they used soybean oil (30%) and aqueous solution (70%), adding whey protein isolate (0.2%), modified starch (0.5%), and composite gum (0.2%). More recently, to replace the animal fat in sheep meat sausages, Santos-Lima et al. [103] elaborated a pre-emulsion of linseed oil with isolated soy protein in a proportion of one part oil and two parts isolated soy protein.

### 3.2. Encapsulation

An alternative to pre-emulsification consists in encapsulating the oils. The encapsulation provides several advantages when they are added to food matrices. For instance, the encapsulating technique allows the masking of off-flavors, reduces lipid oxidation, and eases handling and precision when they are incorporated [78,104]. Oil encapsulation can be classified in several ways based on the chemical and physical transformations of the walls into capsules [105]. Some of the techniques used for the encapsulation of oils are spray-drying, freeze-drying, coacervation, and, to a lesser extent, the use of external ionic gelation and nanoliposomes [106]. The spray-drying process of oils has been the most used technique to obtain particles sized lower than 40 µm. This technique involves the oils’ dispersion into a polymeric solution and a posterior atomization and dehydration, forming microparticles [104,107,108]. The freeze-drying encapsulation consists of vacuum dehydration before freezing the samples for subsequent incorporation in food matrices [109,110]. Coacervation is one of the most common methods to encapsulate oils. In this method, 1 to 10% of the polymer (coacervate) is dissolved in water and the oil is dispersed into a solution at 40–50 °C. The coating (liquid polymer) is deposited and stabilized in the oil phase [111]. Ionic gelation is the most popular extrusion process, with alginate and calcium interaction used for gellification. Nanoliposomes are made by phospholipids as a wall material and have a diameter lower than 100 nm. Thus, Venturini et al. [109] elaborated cookies with 30% fat replacement using chia oil with sodium caseinate and carnauba wax as wall materials by applying freeze-drying as the encapsulation methodology. Ullah et al. [104] carried out a study where they elaborated microcapsules of chia oil utilizing chitosan as the encapsulating material by means of spray-drying with its subsequent application in butter. In the same sense, Ojagh and Hasani [107] investigated the effect of fish oil encapsulated in nanoliposomes using wall materials of lecithin and sunflower oil. Heck et al. [91] studied the microencapsulation of chia oil enriched with rosemary in sodium alginate and CaCl_2_ using ionic gelation as the encapsulating technique (sodium alginate).

### 3.3. Gelled Emulsions

A gelled emulsion is an emulsion with a gel-like network structure and solid-like textural properties [112]. In this type of structure, the emulsions and gel co-exist and have stable rheological properties due to their similarity to animal fat in properties and characteristics [113]. Another important property is that these structures allow the inclusion of both hydrophobic and hydrophilic functional ingredients.

For the elaboration of gelled emulsions, an emulsifier is necessary, which could be of a polysaccharide nature (xanthan gum, konjac matrix, arabic gum, carrageenan, dietary fibre, etc.), a protein nature (sodium caseinate, soybean protein isolate, whey protein isolate, etc.) or a polysaccharide–protein combination [114]. The purpose of the emulsifier is to bind together the water-soluble matrix with the hydrophobic substances, creating water in oil (W/O) or oil in water (O/W) emulsions. Another main component is a gelling agent (pectin, gelatin, gellan gum, algiate methylcellulose, inulin, etc.), which creates the necessary network structure to enable gelation to take place [112].

In the scientific literature, there are several different combinations of emulsifiers, gelling agents, and oils used in the development of gelled emulsion [13,82,84]. In this sense, de Souza Paglarini et al. [115] elaborated a gelled emulsion made with soybean oil (50%), soy protein isolate (4%) as an emulsifier, inulin (16.5%), and water (29.5%) to be utilized as a fat replacer in bologna sausage. Öztürk-Kerimoğlu et al. [116] obtained a gelled emulsion with a 46.8% mixture of peanut oil and linseed oil (10:1) and 3.2% of polyglycerol polyricinoleate as an emulsifier and 50% of the aqueous phase with 40.3% water, 5% lyophilized powder of albumin egg and gelatin, 4% inulin, and 0.7% microbial transglutaminase. Botella-Martínez et al. [21] prepared gelled emulsions to be used as fat replacers in beef burgers based on chia oil or hemp oil (40%) with amaranth flour (10%) as an emulsifier and gellan gum and gelatine (3%) as gelling agents. Lee et al. [117] elaborated a gelled emulsion of canola oil (40%) using k-carrageenan (0, 0.5% *w*/*w*) and methylcellulose (0, 3% *w*/*w*) as gelling agents; the emulsifiers selected were tween 80 (1% *w*/*w*) and polyglycerol polyricinoleate (5% *w*/*w*). A more recent study carried out by Khan et al. [84] generated double water in oil-in-water (W/O/W) emulsions gelled with sunflower oil, whey protein isolate (WPI, as an emulsifier), pectin, and L-ascorbic acid (as gelling agents).

### 3.4. Oleogels

Oleogels are liquid oils turned to be semi-solid structures with viscoelastic properties and hydrophobic natures due to the establishment of a three-dimensional oleogelator network [118]. Oleogel formation allows a high concentration of liquid oil (>90%) to be structured into a “gel-like” system [119]. These new oil structures can be obtained directly by adding the oleogelator to the oil to be structured or indirectly by developing an emulsion beforehand and then dehydrating this structure to form the oleogel [83,120]. Several compounds can be used for oleogelators; the most widely utilized in food products include lipid-based gelators (bee wax, rice bran wax, candelilla wax, fatty alcohols, etc.) [121] and polymers (chitosan, ethylcellulose, hydorxipropilmetylcellulose, methylcellulose, etc.) [122]. Other compounds used as oleogelators include sphingolipids, tocopherols, phytosterols, and lecithin [123].

As regards direct gelation methods, the lipid-based gelators are widely used to elaborate the oleogels [83,120,124,125]. Therefore, in a study carried out by Malvano et al. [120] to replace the butter in the preparation of sponge cake, an oleogel was made by dissolving beeswax at 3 g/100 g in olive oil (97 g/100 g) at 85 °C and continuously agitating it in a vortex at 100 rpm. After that, the blend was cooled at 25 °C until the oleogel was formed. Similarly, Zbikowska et al. [125] made oleogels by dissolving different concentrations of beeswax (2, 4, 6 and 8 g/100 g) in peanut oil at 80 °C. This mixture was then cooled until the oleogel was obtained. In the study conducted by Dent et al. [83], rice bran wax was added as an oleogelator in different proportions (2, 6, and 10 g/100 g *w*/*w*) to corn oil at 90 °C with or without added curcumin. Then, after complete dissolution, the mixture was cooled to obtain the oleogel. Pintado and Cofrades [126] showed how to obtain an oleogel based on a mixture of olive oil and chia oil for application in a fermented dry sausage with 10% beeswax as an oleogelator under constant stirring at 65 °C. More recently, in a study carried out by Oliveira et al. [127] an oleogel system was prepared using phytosterols and lecithin in various ratios (5:5; 6:4; 7:3; 8:2) dispersed in sunflower oil at 85 °C for 30 min under magnetic stirring.

In reference to indirect methodologies used to elaborate oleogels, Wang et al. [128] investigated through two indirect methodologies the preparation of oleogels with hydroxypropylmethylcellulose and methylcellulose as oleogelators and sunflower oil. In the emulsion, the oil content was 18, 33, and 47%, combined with 1.5% cellulose, and then the emulsions were dehydrated at 60 °C for 48 h, resulting in final concentrations of 92, 96, and 97%. For the foam template, 6 g of cellulose was dissolved in 94 g of water at 85 °C and the samples were freeze-dried. A final concentration of 92, 96, and 97% of oil was achieved by adding sunflower oil.

## 4. Food Application

### 4.1. Incorporation of Structured Healthier Oils in Meat Products

Improvements in the lipid profiles and quantity of meat products should be seen by the meat industry as an excellent opportunity for the development of functional products promoting health, differentiation, and competitive advantages. The animal fat of processed meat products is, as mentioned above, one of the ingredients that consumers are most concerned about due to its link with negative health implications (including obesity, cardiovascular diseases, and other diseases in modern society) [129,130,131]. The reformulation of processed meat products to develop heathier meat foods is focused mainly on modifying the lipids of meat products (reduction of total fat, reduction of total cholesterol intake, and improved fatty acid profile) [132,133]. Vegetable oils have been used to achieve reformulation strategies and to develop functional meat products based on lipid profile modification (lower content of saturated fatty acids, higher amounts of mono- and polyunsaturated fatty acids) of processed meat products [134].

Recent studies have shown that the partial and total replacement of animal fat with structuring vegetable oils is an efficient strategy to improve high-fat foods, especially with regard to meat product reformulation with healthier lipids [17,129,135].

It is known that animal fat plays a significant role in the functional characteristics of meat products, such as stability, heat transfer, and texture [13,136], and in the sensory properties (color, taste, elasticity, viscosity, and hardness) [137]. Pork backfat (animal fat widely used in the processing of meat products) is a semi-solid ingredient, but when it is directly replaced by oils, some undesired changes take place, including increased lipid oxidation, changed texture properties, and reduced water holding capacity [14,134]. As mentioned above, meat products’ reformulation, such as the use of healthier oils using several strategies to simulate animal fat properties [130], is in focus. The encapsulation of oils in different matrices [79,89] and the use of oleogeles [25,138] and structured emulsions (gelled emulsions or emulsions gel) [139,140] have been reported as viable strategies which are able to stabilize and structure liquid oils in a semi-solid system comparable to animal fat characteristics and thus avoid negative impacts on final products [130,134].

Several researchers have reported the effect of the reformulation of meat products using structured vegetable oils in fresh, cooked, and dry-cured meat products as a good strategy to replace and improve fat content (quantity and type) [14,139,140,141]. In these studies, the vegetable oils used to replace animal fats were sunflower, soybean, olive, perilla, and chia oils, among others, which improved fatty acid profiles, reducing the level of saturated fatty acids (SFA) and increasing the level of polyunsaturated fatty acids, but also accelerated lipid oxidation reactions and reduced shelf life, in addition to reducing the loss of sensory properties [141].

Table 3 shows different studies that apply vegetable oils through different strategies as animal fat replacers to improve the fat content (quantity and type) of several reformulated fresh meat products.

#### 4.1.1. Fresh Meat Products

Among the fresh meat products, burgers are the most studied meat products due to the importance of their intake among young people. Most of the studies are focused on reducing burgers’ usual content of animal fat (up 20–30%) or replacing the animal fat content with healthier oil [140]. However, the use of vegetable oils in these meat products just affects the technological and sensory properties and shelf life (increased lipid oxidation); therefore, it would be advisable to use different strategies, such as microencapsulation, oleogeles, and gelled emulsions. In fresh meat products (patties, fresh sausages, etc.), the appearance and structure of fat replacers are more important and they can influence visual sensory properties [151].

Microencapsulation allows the incorporation of vegetable oils with high ω-3 polyunsaturated fatty acids. Heck et al. [142] replaced 50% of the animal fat by encapsulated chia and linseed oils by using external ionic gelation and concluded that the microencapsulation was an effective strategy to produce healthier burgers (low fat, higher content of healthier polyunsaturated fatty acids/saturated fatty acids and as ω-6/ω-3 ratios) [80]. Natural antioxidants are recommended when chia oil is used to avoid further lipid oxidation [143]. Recent studies have shown microencapsulation as a novel strategy for the protection of ω-3 polyunsaturated fatty acids from liquid oil against lipid oxidation [78].

Other strategies for the reformulation of burgers with healthy properties are gel emulsions, which show higher stability against oxidation and acceptable sensory properties [152]. Lucas-González et al. [139] evaluated the partial replacement of animal fat by an emulsion gel prepared with chestnut flour and chia oil in the reformulation pork burger, concluding that there was no negative impact on pork burger quality (except for the increase in lipid oxidation, as would be expected), and that the health properties of the reformulated healthy burgers were significantly improved (atherogenicity and thrombogenicity indexes). In two different works, Botella-Martínez et al. [21,153] used gelled emulsions based on chia and hemp oils as partial (25% and 50%) fat replacers in beef burgers during frozen storage and reported better nutritional quality than the control, mainly due to the increase in polyunsaturated fatty acids (specifically α-linolenic (C18:3) and linoleic (C18:2) fatty acids) and the decrease in saturated fatty acids. The burger showed no negative impact during frozen storage. Pintado et al. [144] used emulsion gels with olive oil prepared with chia flour or oat bran as animal fat replacers in reduced-fat fresh sausages, obtaining final meat products suitable for “reduced fat content” and “energy-reduced” claims without strongly affecting the sensory and technological properties. Martínez et al. [23] concluded that chorizos reformulated by replacing pork fat with emulsified seed oils from seeds (50%, 75%, and 100%) presented a better fatty acid profile and were positively evaluated in all the parameters studied.

#### 4.1.2. Emulsified Meat Products

Current studies have concluded that pork backfat substitution by different vegetable oil inclusion strategies in emulsified meat products is a viable option for nutritional enhancement, decreased cholesterol, and a lowered atherogenicity index, thrombogenicity index, and ω-6/ω-3 ratio of cooked meat products [146]. Several authors reported the difficulty of incorporating vegetable or marine oils rich in ω-3 polyunsaturated fatty acids in the heat treatment of meat products due to the higher degree of oxidation and technical and sensorial complications in the final product [91,136,137,154]. Likewise, the composition of the oil and the fat: protein ratio could affect the quality of the final products because they affect the emulsion stability [137].

Different effects have been reported in reformulated cooked meat products, such as improved lipid profile, softer texture through the increase of unsaturated fatty acid content [89], a better dispersion and distribution of the vegetable oils [89,155], and increased yellowness values due to the effect of vegetable oils [141,156]. Gelled emulsions with olive oil, chia oil, hemp oil, soybean oil, or linseed oil have been used as animal fat replacers in cooked products with an optimization in the lipid profile either through an increase in the monounsaturated fatty acids content, e.g., when olive oil is used [126], or through the increase in levels of polyunsaturated fatty acids, e.g., hemp oil [136] or chia oil [89]. Botella-Martínez et al. [136] studied the incorporation of gelled emulsion prepared with hemp oil and buckwheat flour in sausages at different percentages of substitution of pork fat (25%, 50%, 75%, and 100%). The reformulated frankfurters exhibited an improved lipid profile, without affecting the technological properties and lipid oxidation, despite the high PUFA content in hemp oil. Several authors have attributed this to the capsulated oil droplets in the gel matrix, which would act as a protective barrier against oxidation [156]. In other studies, an increase in lipid oxidation was observed in vegetable oils with a high content of ω-3 polyunsaturated fatty acids (e.g., chia oil), but vegetable oils rich in monounsaturated fatty acids did not show this increase [89,156]. De Souza Paglarini et al. [145] replaced the pork backfat of reduced-fat frankfurters with emulsion gels formulated with soybean oil, sonicated soy protein isolate dispersion, inulin, and carrageenan. They concluded that reformulated frankfurters could be considered a good source of fiber and as high in unsaturated lipids; however, some of the sensorial properties were affected (juiciness, soft texture).

Several works have studied the substitution of animal fat with different structured vegetable oils in the development of Frankfurt sausages as a viable strategy for developing healthier meat products [24,25,26,98]. Barbut and Marangoni [26] observed a reduction in hardness, which may be due to different interactions between the vegetable oil organogels within the meat matrix system. They concluded that the type of fat/oil and its degree of saturation play a major role in determining the hardness/texture of the final meat product. Da Silva et al. [98] developed an oleogel with pork skin, water, and high oleic sunflower oil. They showed a significant reduction in cooking loss and an increase in emulsion stability when the backfat was replaced by this oleogel in Bologna, in contrast to other works where oleogels were used as fat replacers. They reported that this optimum impact of technological properties may be due to the pork skin collagen, which interacts with proteins, developing a more rigid gel matrix and preventing the exudation of water and fat from the meat system during cooking [24,98].

#### 4.1.3. Traditional Fermented Meat Products

The improvement in the lipid profile has promoted the reformulation of traditional meat products. Most studies have focused on the replacement of animal fat by non-fat ingredients (inulin, cellulose gel, konjac gel, or boiled quinoa) or vegetable oils (olive, soybean, linseed, grapeseed) as liquid, encapsulated, or pre-emulsified fat [25,150,157]. It should be emphasized that fat is a critical ingredient in the development of the flavor, texture, and juiciness of dry-cured sausages [158,159].

The direct replacement by healthier oil or integration by vegetable oil in water emulsion systems to replace animal fat is not technologically viable because of technological difficulties and its negative impact on texture and sensorial attributes [160,161]. New strategies are required to preserve the textural properties of the final product, especially considering the complex ripening process and the strong role of fat in moisture loss during the drying process [93]. At present, studies are directed towards the incorporation of vegetable oils into fermented sausage in edible structured gels (oleogeles) or encapsulated forms.

Stajić et al. [150] replaced 20% of the backfat of dry fermented sausages with grapeseed oil prepared as encapsulated and reported changes in the texture characteristics (lower hardness, chewiness, and cohesiveness) which were perceived as negative characteristics in a sensory evaluation. Alejandre et al. [93] substituted different levels of animal fat by a gelled emulsion of linseed oil in dry fermented sausages during the chopping step. The reformulated dry sausages showed a small defect in the appearance of the slices and did not show oxidation problems. In addition, from a sensorial point of view, no perceptible differences for taste and juiciness were obtained.

Recently studies have shown the potential of the edible oleogels elaborated with vegetable oils to be used as substitutes for pork backfat in fermented sausages [126,148]. This substitution provides an improvement of the polyunsaturated fatty acids/saturated fatty acids ratio and ω-6/ω-3 ratio, although the impact was negative for sensory properties. Franco et al. [148] studied the oleogels of linseed oil based on mixtures of γ-oryzanol, β-sitosterol, and beeswax for the replacement of pork backfat in fermented sausages. They concluded that the drying process was dependent on the oleogelator and the level of replacement, affecting textural and sensorial attributes. The fermented sausages with beeswax oleogel presented a higher moisture content due to lower drying speed, which indicates that oleogel based on beeswax would behave like a barrier, avoiding water loss during the drying process. Pintado and Cofrades [126] concluded that oleogels or emulsion gels, elaborated with a chia and olive oil mixture, were optimal strategies for the development of dry healthier fermented sausages with an improved lipid profile. They recommended that more studies should be carried out to improve the negative impacts on the sensory characteristics of meat products due to the fact that fat is a basic component since it contributes to technological and sensory properties (softness and juiciness), water holding capacity, emulsion stability, and shear strength [17].

### 4.2. Incorporation of Structured Healthier Oils in Dairy Products

Milk is a complete and balanced food as a source of nutrients [162] but it also contains a considerable amount of saturated fat. This high fat content has been the subject of various reduction strategies to diminish potential health risks. Moreover, there is a trend towards the consumption of plant-based foods, which are analogues of many dairy products, by the total or partial substitution of dairy components, mainly fats [163,164,165].

In addition to the reduction of milk fat content, products have also been developed where the lipid fraction of dairy products has been replaced or enriched. One of the most commonly used methods has been microencapsulation, which shows great potential for the protection of bioactives or vegetable oils incorporated during processing, storage, and transportation [163]. Substituting or enriching the lipid fraction of dairy products by integrating functional chia and sunflower oils in spray-dried emulsions is a suitable way to incorporate these ingredients to increase their added value [166]. Both the high nutritional value and the expensive price of dairy products have encouraged one of the most common frauds: replacing milk fat with cheaper vegetable oils due to the economic advantage of such substitution. Dairy fat may be replaced or mixed with cheap oils such as palm, sunflower, and corn oils [167,168,169]. Several research works assess the effects on quality parameters of dairy products with total or partial substitution of milk fat by vegetable oils with different incorporation strategies, as shown in Table 4. The most frequently modified dairy products are cheese, yoghurt, and ice cream, which coincide with those with the highest consumption.

#### 4.2.1. Cheese

Various studies have been carried out modifying different types of cheese by including vegetable oils as dairy fat replacers in order to alter their sensory and nutritional attributes and modify their lipid profiles [171,183,184]. The resulting cheese has less cholesterol and different a composition of saturated and unsaturated fatty acids. The inclusion of vegetable oils in cheese has become a good opportunity to increase unsaturated fatty acids [185] and to improve antioxidant properties with effects on sensory attributes [171]. In most cheeses, milk fat is one of the major components, which results in many consumers having to limit their consumption for health reasons [184]. The incorporation of vegetable oils, such as high oleic sunflower oil, could help to solve this problem [186]. In a study carried out by Bermúdez-Aguirre and Barbosa-Cánovas [187], cheese was fortified with ω-3 fatty acids obtained from vegetable sources (canola, flaxseed, and soybean). These authors found that the reformulated cheese had some differences in texture but had good scores in sensory evaluation. Achachlouei et al. [183] replaced dairy fat in white brine cheese by half or total substitution with emulsified canola and olive oils. The cheese containing these vegetable oils had lower amounts of saturated fatty acids and higher amounts of unsaturated fatty acids compared with the original cheese and also received better sensory scores. The substitution of milk fat with palm oil decreased dry matter, increased texture parameters, and decreased sensory quality in a white brine cheese, as reported by Sulejmani et al. [188].

Vegetable oils, such as soybean oil, palm oil, peanut oil, canola oil, coconut oil, and corn oil, are also used in the elaboration of most processed cheeses. Processed cheeses are products elaborated from cheeses unmarketable due to defects in shape or ripeness; they are produced to reduce costs and food waste and can meet special dietary needs through changes in their formulation [184,189].

Processed cheeses (which cannot be called cheese as they are not produced solely from milk, milk protein, milk fat, or other milk solids) can be classified as:

- Analogue or imitation cheese, made from dairy and/or nondairy ingredients, formulated with specific nutritional and/or functional properties, according to consumer needs [184,189]. In imitation cheese, the fat source can come from soybean oil, rapeseed oil, palm oil, canola oil, sunflower oil, and corn oil [165,186].
- Functional processed cheese, made from dairy and/or nondairy ingredients and fortified with some bioactive compounds with functional properties.
- Plant-based processed cheese, made from nondairy ingredients [189].

As important as the type of vegetable oil incorporated is the mode of incorporation. Garbin Cardoso et al. [170] reported that microencapsulation by ionic gelation, without the need for heating, is a technology used to stabilize vegetable oils. The results of their study, which incorporated microencapsulated chia oil in a processed cheese from Grana Padano and mozzarella cheese, showed that microencapsulation was able to mask the oil taste.

#### 4.2.2. Yoghurt

The incorporation of vegetable oils into yoghurt can be due to various reasons, such as improving the lipid profile of the dairy product or using the oil as a transporter for another ingredient through the formation of emulsions or microencapsulation. Izadi et al. [173] used soybean oil to create an oil-in-water emulsion to produce an enriched yoghurt with phytosterol as a functional ingredient due to its cholesterol-lowering properties. The formation of this emulsion avoids a chalky taste due to phytosterol enrichment. The results showed no significant differences between the enriched yoghurt and the control with regard to texture, appearance, flavor, and overall acceptance; also, the enriched yoghurt had lower syneresis, higher firmness, and lower apparent viscosity. In a study carried out by El Sayed et al. [174], stirred yoghurt was fortified with two models of microcapsules based on synbiotic extra virgin olive oil (EVOO). The results showed that the addition of nanoemulsion microcapsules to yoghurts increased antioxidant activity more than in plain yoghurt. Pequi (*Caryocar coriaceum* Wittm.) oil was also incorporated due to its high levels of unsaturated fatty acids. To avoid its strong taste, the microencapsulation method was employed, which also protects against oxidation [175]. Li et al. [176] applied whey protein emulsion gel microparticles from corn oil to yoghurt as a fat substitute, finding that there was an improvement in textural and rheological properties, water holding capacity, and storage stability, while the sensory defects associated with fat reduction were reduced. When palm oil was incorporated in the mixture of ingredients to make yoghurt, the yoghurt obtained showed equal sensory attributes and retained probiotic properties [190].

#### 4.2.3. Ice Cream

The fact that ice cream is one of the most popular types of dairy products worldwide and the concern about the effect of the food on health have both led to an increase in demand for lower calorie or better lipid profile ice creams, alongside the consideration that these modifications must not spoil the sensory attributes and storage stability of ice cream [178,179,181]. Among the strategies carried out to achieve the substitution of milk fat is the incorporation of vegetable oils, which in some cases happen to be by-products of other agri-food processes. This is accompanied by nutritional benefits and, sometimes, as in the case of the use of oleogels, the improvement of some technological characteristics, such as greater overrun, with enhancements in texture and appearance [178,179]. Paul et al. [180] investigated the incorporation of basil oil microcapsules in ice cream. They found that sensorial attributes were not affected and the antioxidant properties were improved due to the polyphenolic content; meanwhile, the direct use of basil oil has been found to cause adverse effects in sensorial and antioxidant properties. However, when extra virgin olive oil (EVOO) is incorporated in the mixture of ice cream ingredients, the ice cream matrix masks EVOO bitterness and there is a higher content of biophenols. Güven et al. [182] demonstrated that similar or even better quality characteristics were obtained when the dairy fat of ice cream was replaced with hazelnut oil and olive oil.

#### 4.2.4. Butter

More than 82% of butter is fat, comprising mostly saturated fat with good technological properties. However, the consumption of butter in excess is associated with health problems [191]. In addition to this association, the environmental impact butter generates as a food of animal origin has led to the development of alternatives of vegetable origin, such as echium oil or extra virgin olive oil. Gutiérrez-Luna et al. [164] have developed gel emulsions from both oils that could be used as potential butter analogues, both for their technological properties and their nutritional value, although more in-depth studies are needed for their full development and commercial use.

## 5. Conclusions

This review highlights the interest of the scientific community in reducing the saturated fat content of meat and dairy products and replacing it with vegetable and marine oils with healthier fatty acid profiles. It shows how the different strategies used to structure these oils affect their mode of application and how the chemical composition, techno-functional, physicochemical, and sensory properties of the reformulated products are modified.

As a future perspective, more research should be carried out in two directions. On the one hand, the protection of these polyunsaturated oils from oxidative processes should be investigated, as they can lead to changes in the organoleptic properties that occur during the production process of meat and dairy products. On the other hand, more research has to be performed to know the bioaccessibility and bioavailability of these fatty acids when they are ingested and to know the possible interactions with other components of the food matrix that could affect or promote their release and/or absorption.

## Figures and Tables

**Table 1:** Lipid profile of several vegetable and animal oils.

		Oils from Oilseeds					
	Saturated Fatty Acids	Monounsaturated Fatty Acids		Polyunsaturated Fatty Acids			Ref.
		Oleic Acid	Other	Linoleic Acid (*w-6*)	Linolenic Acid (*w-3*)	Others	
Soybean	15.6–16.2	21.4–22.6	0.2–1.0	52.0–54.5	10.0–12.8	0.8–1.2	[29,30]
Canola	7.4–8.2	61.8–63.5	1.5–2.4	18.6–20.2	9.1–10.4	0.4–0.7	[31,32]
Sunflower	15.5–17.0	22.4–23.3	0.2–0.8	55.0–57.8	3.6–4.2	0.9–1.4	[33,34]
Cottonseed	25.9–26.8	17.8–18.6	1.2–1.8	52.1–54.0	0.6–1.0	0.1–0.5	[35,36]
Corn	15.6–17.0	25.3–26.7	0.3–0.5	52.0–53.4	1.0–1.2	4.4–4.9	[37,38]
Peanut	13.5–15.4	53.0–54.2	0.3–0.6	29.8–32.0	0.2–0.4	0.8–1.0	[39,40,41]
Chia	8.0–8.5	4.0–4.8	---	20.0–22.7	68.0–69.5	---	[42,43]
Hemp	7.0–7.8	8.3–9.0	---	52.6–54.0	21.7–22.0	8.0–8.6	[44,45]
Date	44.5–47.0	42.6–45.0	---	8.0–8.6	n.d.	n.d.	[46]
		**Oils from fruits**					
Olive	11.1–12.7	72.3–78.6	1.0–1.5	8.8–9.1	0.7–0.9	5.1–5.5	[31,47,48]
Coconut	80.0–82.5	6.0–7.8	5.0–6.3	---	---	1.7–2.1	[49,50]
Avocado	9.60–12.5	58.6–61.5	8.7–10.5	9.0–12.5	1.5–2.4	1.5–1.9	[31,51]
		**Oils from marine origin**					
Seaweed	50	11.2	11.2–12.0	5.0–5.8	1.5–1.8	26.5–28.0	[52,53,54]
Fish (*Sardina pilchardus*)	32	25	7.2–7.8	3.5–4.2	1.0–1.2	29.5–31.3	[55,56]
		**Oils from Insects**					
*Tenebrio mollitor*	33.4	35.8	2.1	22.8	0.1	5.8	[57]
*Acheta domesticus*	31.2	20.2	0.8	41.4	1.1	5.3	[58]

**Table 2:** Technological strategies to structure vegetable and marine oils.

Structure Components	Structuring Strategy	Procedure	Application	Ref.
- Sunflower oil (47 g/100 g) - Water (51 g/100 g) - Cellulose ethers (2 g/100 g)	Pre-emulsification	First, the cellulose ether was dispersed in the oil and then the water was gradually added. Finally, the mixture was homogenized until the emulsion was obtained.	Cocoa creams	[85]
- Soy protein - Water - Rapeseed oil - Ratio (1:4:4)	Pre-emulsification	Initially, soy protein was added into the water and mixed. Then, the oil was added gradually in a bowl chopper fitted with three blades and operating at two speeds.	UK-style sausages	[86]
- Whey protein powder - Hazelnut oil - Ratio (1:2)	Pre-emulsification	The whey protein was mixed with hazelnut oil with a hand blender until the emulsion was obtained.	Sucuk Turkish fermented sausages	[87]
- Fish protein isolate (4%) - Soybean oil	Pre-emulsification	Fish protein isolate was dissolved in phosphate buffer (10 mM, pH 7). The solution was mixed with soybean oil, in equal amounts (oil volume fraction 0.5), using the homogenizer at 19,000 rpm for 5 min.	Pork sausages	[88]
- Poppy oil or chia oil 100 mL - Maltodextrin 22 g	Pre-emulsification	Oil and maltodextrin were mixed using a spoon and kept at 4 °C for 24 h.	Fresh chorizo	[23]
- Water (650 mL) - Tiger nut oil (50 g) - Lactose (50 g) - sodium caseinate (50 g)	Encapsulation	Dry spryer conditions were: feed rate 30%, inlet temperature 145 °C, and the aspirator at 80%.	Deer pâté	[89]
- Fish oils (1 kg) - Water (12 kg) - Maltodextrin (1.95 kg) - Gum arabic (0.9 kg) - Caseinate (0.15 kg)	Encapsulation	Dry spryer conditions were: feed rate 75 L/h, inlet temperature 180 °C, and outlet temperature 80 °C. Drying process lasted 3 h.	Frankfurters	[90]
- Chia oil - Sodium alginate solution (2%)	Encapsulation	External ionic gelation technique.	Pork burgers	[91]
- Fish oil 10% - Lecithin 3% - Chitosan 2%	Encapsulation	Dry spryer conditions were: feed rate 1 L/h, inlet temperature 180 °C, and outlet temperature ranged 85–90 °C.	Pork burgers	[92]
- Linseed oil (40%) - Carrageenan (1.5) - Water (58.5%) - Polysorbate 80 (0.12 g/emulsion)	Gelled emulsion	The oil phase with the surfactant was added to the aqueous phase (water + carrageenan) and homogenized.	Dry fermented sausages	[93]
- Water (56%) - Alginate-based hydrogels (6.7%) - Olive oil (37.3%)	Gelled emulsion	Water and olive oil were mixed for 1 min. Then, gelling agent was addedand homogenized during 3 min and then left to rest for 2 h. After that the mixture was cooled at 4 °C.	Deer fermented sausages	[94]
- Açai oil 200 mL - Water 100 mL - Konjac flour (0.86 g) - Sodium alginate (2 g)	Gelled emulsion	Sodium alginate and konjac flour were dissolved in water at 60 °C with constant stirring. The emulsion (2:1 oil:water) was homogenized in the biocomposite obtained.	Beef burgers	[95]
- Water (45%) - Chia or hemp oils (45%) - Buckwheat flour (9%) - Carrageenans and locust bean gum (1%)	Gelled emulsion	Carrageenans and locust bean gum were dissolved in water at 80 °C with constant stirring. Chia or hemp were added to aqueous solution and homogenized.	Plant-based burgers	[82]
- Sesame oil - Beeswax (10%)	Direct oleogel	Oil and beeswax mixture were heated at 70 °C with continuous stirring until complete dissolution of beeswax.After that the blend was cooled at room T^a^.	Beef burgers	[96]
- High oleic sunflower oil (90%) - Rice bran wax (10%)	Direct oleogel	Sunflower oil was combined with rice brand wax and the mixture was heated at 80 °C and stirred (5 min). After that the blend was cooled at room T^a^.	Ice cream	[97]
- Water - High oleic sunflower oil - Pork skin - Ratio 1:1.5:1.5	Direct oleogel	Water, high oleic sunflower oil, and pork skin (cooked at 80 °C) were mixed in a blender.	Bologna sausages	[98]
- Linseed oil - Mixture of oryzanol and β-sitosterol (8%)	Direct oleogel	Oryzanol and *β*-sitosterol were dispersed under stirring until solubilization in linseed oil at 80 °C for 30 min.	Pork burgers	[99]
- Carnauba wax - Canola oil - Ratio 1:9	Direct oleogel	Carnauba wax and canola oil were heated at 90 °C with continuous agitation. Then the sample was cooled at room T^a^.	Cakes	[100]

**Table 3:** Summary of studies assessing effects on quality parameters of meat products with total or partial substitution of animal fat by vegetable oils with different incorporation strategies.

Meat Products	Vegetable Oil	Incorporation Strategy	Replacement (%)	Response on Meat Product	Ref.
Burger	Chia and linseed oils	Microencapsulation (external ionic gelation)	Replacement at 50%	Improved important technological properties (cooking loss and fat retention). Low fat, higher content of healthier polyunsaturated fatty acids/saturated fatty acids, and ω-6/ω-3 ratio ratios. Acceptable sensory properties.	[142]
Burger	Chia and linseed oils	Hydrogelled emulsion	Replacement at 20, 40, 60, 80, and 100%	Increased protein:lipid ratio. Improved the fatty acid profile of raw burgers. Increased TBARs. Non-affected technological properties.	[80]
Burger	Chia oil	Microencapsulation (enriched with rosemary)	Replacement at 50%	Non-impact on the volatiles profile. Decreased in volatiles from lipid and protein oxidation. Decreased sensory descriptors related to lipid oxidation.	[143]
Burger	Chia and hemp oil	Gelled emulsions (amaranth flour, gellan gum, and gelatin)	Replacement at 25 and 50%	Improved nutritional characteristics of burgers. Non-affected technological or sensory properties. More susceptible to lipid oxidation.	[21]
Fresh chorizo	Melon and pumpkin seed oils	Oil emulsions	Replacement at 50%, 75%, and 100%	Softer texture. Better fatty acid profile, decreased in saturated fatty acids, and increased linoleic and linolenic fatty acids.	[23]
Fresh sausages	Olive oil	Gelled emulsions prepared with chia and oats	Replacement at 90%	Improved fat, minerals, and amino acid contents. Cooking loss was lower. Higher Kramer shear force values. Affected sensory properties, but were judged acceptable.	[144]
**Emulsified Meat Products**					
Frankfurter	Soybean oil	Emulsion gels (EG) prepared, sonicated and non-sonicated soy protein isolate dispersions, carrageenan, and inulin	Replacement at 100% The pork backfat (10 and 20%) was replaced by the gelled emulsion	Good source of fiber. A reduction of 19–29% in energy value. A reduction of 35, 72, and 63% in as ω-6/ω-3 ratio, atherogenic index (AI), and thrombogenic index (TI), respectively.	[145]
Frankfurter	Soybean oil	Oleogels structured with rice bran wax	Replacement at 100%	Less dark and less red. Higher in the essential polyunsaturated fatty acids linoleic (18:2n6) and α-linolenic (18:3n3). Non-negatively influence the technological quality.	[24]
Frankfurter	Linseed oil	Oleogel gelled with beeswax	Replacement at 0% 25% and 50%	The fatty acid profile was substantially improved and saturated fatty acid content, as ω-6/ω-3 ratio and cholesterol were reduced. Increased the yellowness with linseed oleogel. Increased cohesiveness, gumminess, and chewiness.	[25]
Frankfurter	Canola/soy/ flaxseed oil	Oleogels	Replacement of 100%	Higher hardness values. Springiness was lower. Flaxseed oil provided the highest b*. Reduced cooking loss.	[26]
Emulsified sausages	Peanut and linseed oil	Gelled emulsion	Replacement of up to 40%	Healthier lipid composition and improved nutritional ratios: Decreased saturated fatty acids and cholesterol and increased mono and polyunsaturated fatty acids. Improved emulsion stability and cooking behaviors. Alterations in color and texture: higher yellowness and increased the hardness. Decreased oxidative stability.	[146]
Bologna sausage	Soybean oil	Emulsion gels prepared with chia flour and/or soy protein isolate, inulin, carrageenan, sodium caseinate, and sodium tripolyphosphate	Replacement at 50 and 100%	Improved lipid profile of the sausage. Lower fat content. Affected the color of sausages: increased L* and reduced a*. More homogeneous batter and a compact structure. Greater hardness, chewiness, and shear force.	[145]
Deer pâté	Tigernut, chia, or linseed oils	Microencapsulated	Replacement at 50%	Decreased fat and cholesterol contents. Decreased the total amount of saturated fatty acids and increased polyunsaturated fatty acids (chia and linseed pâtés) or monosaturated fatty acids contents (tigernut pâtés). Modification of color parameters. Softer textures.	[89]
Bologna	High oleic sunflower oil	Oleogel prepared with pork skin, water, and high oleic sunflower oil	Replacement at 25, 50, 75, and 100%	Healthier lipid profile: reduction of approximately 10% cholesterol levels.Increased the proportion of oleic acid and decreased the proportion of linoleic acid. Non-changes in the oxidative stability. The acceptance and the sensory profile of the samples were not affected by the substitution of up to 50%. Decrease in cooking loss.	[98]
Pâtés	Mixture of olive, linseed, and fish oil	Oleogels produced with ethyl cellulose and beeswax oleogel	Replacement at 15%	Optimal fatty acid profile from a health standpoint (high polyunsaturated fatty acids/saturated fatty acids ratio and low as ω-6/ω-3 ratio). Emulsion stability, texture, and color of pâtés not affected. Increased lipid oxidation. Sensory attributes similar.	[147]
**Traditional Fermented Meat Products**					
Dry fermented sausages	Linseed oil	Gelled emulsion	Replacement at 26.3%, 32.8%, and 39.5%	Increased polyunsaturated fatty acids supply (up to 10.3%) and reductions in ω-6/ω-3 ratio (75, 82, and 84%, respectively). Non-affected peroxides and Thiobarbituric reactive substances (TBARs) values.	[93]
Dry fermented sausage (*Salchichón*)	Linseed oil	Oleogels produced with 8% ɣ-oryzanol, β-sitosterol, and beeswax	Replacement at 20 and 40%	Improvement of the fatty acid profile. Color and sensory parameters were strongly affected. Quality parameters such as pH and color also changed with the inclusion of oleogels. Changed in the sensory quality.	[148]
Dry fermented sausage	Linseed oil	Gelled emulsion	Replacement at 65%	Lower saturated fatty acids and monounsaturated fatty acids and higher polyunsaturated fatty acids content with an improved as ω-6/ω-3 ratio α- and linolenic acid increment. Decreased in springiness, chewiness, and hardness and increase in adhesiveness. Lower L* and higher a*. Higher susceptibility to oxidation and lipolysis.	[149]
Dry fermented sausage	Grapeseed oil	Liquid, encapsulated, and pre-emulsified	Replacement at 20%	Higher weight loss. Lower hardness, chewiness, cohesiveness.	[150]
Dry fermented meat product (*Fuet*)	Olive and chia oil	Oleogels and gelled emulsion	Replacement at 80%	Improved fatty acid profile. Decrease of ω-6/ω-3 ratio. Emulsion gel as animal fat replacer had similar hardness to the control whereas those with oleogel were softer.	[126]

**Table 4:** Summary of studies assessing effects on quality parameters of dairy products with total or partial substitution of milk fat by vegetable oils with different incorporation strategies.

Dairy Product	Vegetable Oil	Incorporation Strategy	Effect on Dairy Product	Ref.
Processed cheese	Chia oil	Microcapsules versus free oil	Microencapsulation process masked the flavor of the oil.	[170]
Cheddar cheese	Chia oil	Incorporated in mixture of ingredients	No effect on sensory attributes; antioxidant properties improved.	[171]
*Queso blanco* cheese	Flaxseed oil	Incorporated during homogenization, coagulation, or salting	The best scores are obtained when oil is incorporated during homogenization.	[172]
Yoghurt	Soybean oil	Emulsion O/W for dissolving phytosterols as functional ingredient	No significant difference in texture, appearance, flavor, and overall acceptance;lower syneresis, higher firmness, and lower apparent viscosity.	[173]
Yoghurt	Extra virgin olive oil (EVOO)	Microcapsules of EVOO and synbiotic bacteria	Increased antioxidant activity.	[174]
Yoghurt	Pequi oil	Microcapsules of pequi oil	Increase in the percentage of oleic acid; delayed oxidation.	[175]
Yoghurt	Corn oil	Emulsion gel microparticles from corn oil	Improvement in textural and rheological properties, water holding capacity, and storage stability; sensory defects were reduced.	[176]
Yoghurt	Flaxseed	Nanoemulsion	Increase solubility, bioavailability, and protection of ω-3.	[177]
Ice cream	Vegetal oil	Oleogels	Greater overrun, which implies an improvement in texture and appearance.	[178]
Ice cream	Grape seed oil	Incorporated in mixture of ingredients	High nutritional antioxidant activity.	[179]
Ice cream	Basil oil	Encapsulated by Spray-drying	High antioxidant and phenolic content; sensorial attributes were not affected.	[180]
Ice cream	Extra virgin olive	Incorporated in mixture of ingredients	Matrix masked EVOO bitterness; higher content of biophenols.	[181]
Ice cream	Hazelnut and olive oil	Incorporated in mixture of ingredients	Similar or even better quality characteristics.	[182]

## Data Availability

The data presented in this study are available on request from the corresponding author.
